# Impaired vascular-mediated clearance of brain amyloid beta in Alzheimer’s disease: the role, regulation and restoration of LRP1

**DOI:** 10.3389/fnagi.2015.00136

**Published:** 2015-07-15

**Authors:** Anita Ramanathan, Amy R. Nelson, Abhay P. Sagare, Berislav V. Zlokovic

**Affiliations:** Department of Physiology and Biophysics, Zilkha Neurogenetic Institute, Keck School of Medicine, University of Southern CaliforniaLos Angeles, CA, USA

**Keywords:** amyloid β clearance, blood-brain barrier, lipoprotein receptor-related protein 1 (LRP1), *PICALM*, Alzheimer’s disease (AD)

## Abstract

Amyloid beta (Aβ) homeostasis in the brain is governed by its production and clearance mechanisms. An imbalance in this homeostasis results in pathological accumulations of cerebral Aβ, a characteristic of Alzheimer’s disease (AD). While Aβ may be cleared by several physiological mechanisms, a major route of Aβ clearance is the vascular-mediated removal of Aβ from the brain across the blood-brain barrier (BBB). Here, we discuss the role of the predominant Aβ clearance protein—low-density lipoprotein receptor-related protein 1 (LRP1)—in the efflux of Aβ from the brain. We also outline the multiple factors that influence the function of LRP1-mediated Aβ clearance, such as its expression, shedding, structural modification and transcriptional regulation by other genes. Finally, we summarize approaches aimed at restoring LRP1-mediated Aβ clearance from the brain.

## Introduction

Alzheimer’sdisease (AD) is a neurodegenerative disorder characterized by abnormal elevations of amyloid β (Aβ), neurofibrillary tau tangles and neurovascular dysfunction. Aβ is generated from the transmembranous amyloid precursor protein (APP) after proteolytic cleavage by β- and γ-secretase (Selkoe, 2001) and is proposed to have multiple roles in the brain such as, to name a few, neurotrophic activity, modulation of synaptic plasticity, neurogenesis, metal ion sequestration, antioxidant activity, and calcium homeostasis (del Cárdenas-Aguayo et al., 2014); and also interferes with signal transduction within the neurovascular unit affecting multiple neurovascular functions (Zlokovic, 2011). Thus, regulated clearance of Aβ via receptor-mediated transport potentially allows for controlled regulation of all these Aβ functions at any given time and also prevents accumulation of excess Aβ as a metabolic waste product.

Neurotoxic accumulation of Aβ in the brain has been hypothesized to result from an imbalance in the Aβ homoeostasis i.e., between its production and clearance (Zlokovic, 2011; Musiek and Holtzman, 2015). Recent evidence in humans has suggested that indeed faulty Aβ clearance mechanisms, but not overproduction, contribute to pathological accumulations of cerebral Aβ in late-onset sporadic AD (Mawuenyega et al., 2010). Because impaired clearance of Aβ is now widely identified as a contributing factor towards AD progression, studying Aβ clearance mechanisms, their regulation and devising methods to modulate clearance function is of importance in generating potential AD-related therapeutic interventions (Tarasoff et al., in press).

To prevent Aβ aggregation and deposition, various physiological clearance mechanisms exists to help steer Aβ removal from the brain, namely, transvascular clearance across the blood-brain barrier (BBB; Zlokovic, 2008, 2010, 2011), interstitial fluid (ISF) bulk flow i.e., traditional perivascular clearance (Weller et al., 2008; Hawkes et al., 2012) and glymphatic paravascular clearance (Iliff et al., 2012), cerebrospinal fluid (CSF) absorption (Pollay, 2010) and enzymatic degradation (Farris et al., 2003; Kanemitsu et al., 2003; Hernandez-Guillamon et al., 2015). Aβ removal from brain by transcytosis is typically a concentration-dependent process physiologically driven by a much higher brain-to-plasma concentration gradient of Aβ This receptor-mediated mechanism allows for removal of Aβ from both the mammalian (Zlokovic et al., 2010) and human (Roberts et al., 2014) brain across the largest possible surface area for removal of about 100 cm^2^ of the capillary endothelium per gram of brain tissue representing the surface of the BBB *in vivo* (Zlokovic, 2011). Previous studies have demonstrated that the majority of Aβ is cleared by the BBB (~85%) under physiological conditions with a smaller percentage being cleared by ISF bulk flow (Shibata et al., 2000; Zlokovic and Frangione, 2003). Disrupted Aβ transcytosis leads to accumulation of Aβ in the brain causing pathophysiologal changes as shown by multiple previous studies. In this review, we focus on transvascular clearance of Aβ across the BBB mediated by low-density lipoprotein receptor-related protein 1 (LRP1) in association with AD.

### Blood-Brain Barrier and LRP1

Maintenance of a toxin-free brain microenvironment is essential for normal neuronal function. The endothelial cells of brain capillaries are compactly connected end-to-end by tight junction proteins forming the BBB, isolating the brain ISF-CSF compartments from the plasma compartment and authorizing entry of molecules from the blood into the brain (Zlokovic, 2008; Abbott et al., 2010). The specialized brain vascular endothelial cells of the BBB, along with pericytes, glial cells and neurons form the neurovascular unit (NVU; Zlokovic, 2011). This arrangement is very different from the highly permeable and lenient systemic capillaries permitting transport of solutes and bigger molecules into the parenchymal tissue space (Mann et al., 1985). At the level of brain capillaries, while oxygen from the blood diffuses freely into the brain, the entry of polar molecules is typically restricted by the BBB (Zlokovic, 2011). However, nutrients can cross the BBB using specific transporters expressed within the brain endothelium (Zlokovic, 2011). The polarized distribution of transporters on the luminal and abluminal endothelial membranes of the BBB allows the brain endothelial cells to control movement of ions, nutrients and other molecules between brain and blood in a highly-regulated manner to match metabolic demands of brain. Although, larger molecules are typically excluded from the brain by the BBB, some proteins and peptides can still cross the BBB slowly if their respective transporters and/or carriers are expressed within the brain endothelium (Zlokovic et al., 1985, 1987; Zlokovic, 1995; Mackic et al., 2002).

At the BBB, the transporter LRP1 plays a pivotal role in maintaining Aβ homeostasis in the central nervous system (CNS). LRP1 is expressed mainly at the abluminal side of the BBB (Zlokovic, 2008; Ueno et al., 2010; Zhao et al., 2015). LRP1 is the primary receptor mediating transport of Aβ across the BBB into circulation, thereby clearing it from the brain. LRP1 is a member of the LDLR family. This cell surface receptor is highly expressed on multiple cell types (Kanekiyo and Bu, 2014). At the neurovascular interface, LRP1 is expressed by vascular endothelial cells forming the BBB, vascular mural cells, namely, pericytes and vascular smooth muscle cells (VSMCs), neurons and astrocytes (Zlokovic et al., 2010; Sagare et al., 2012; Kanekiyo and Bu, 2014).

The distinct structural arrangement of LRP1 enables its multifaceted role as a cargo transporter, a multifunctional scavenger and a signaling receptor involved in endocytosis of its ligands (Lillis et al., 2008). LRP1 recognizes and is involved in the endocytosis of more than 40 different ligands including apolipoprotein E (apoE), APP and Aβ (Zlokovic et al., 2010; Sagare et al., 2012; Kanekiyo and Bu, 2014).

**Figure 1:**
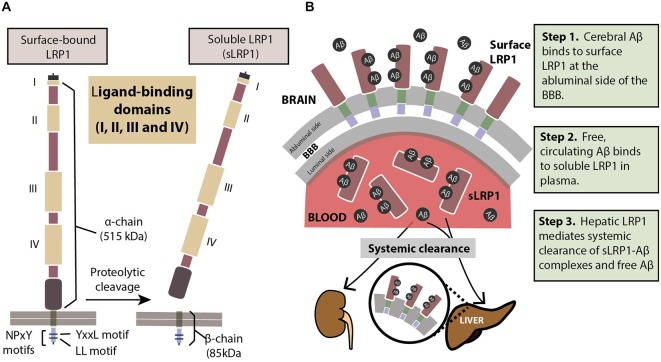
**(A)** The structure of LRP1. The extracellular domain consists of a heavy α-chain (515 kDa) with four ligand-binding domains. The light chain or the β-chain (85 kDa) extends into the intracellular compartment where it has two characteristic NPxY motifs involved in endocytosis and cell signaling. Proteolytic cleavage of surface-bound LRP1 yields the soluble sLRP1. **(B)** The three steps of LRP1-mediated Aβ clearance from brain (*step 1*), from blood (*step 2*) and from the body (*step 3*). For description of these three steps please see panel **(B)**. See “Role of LRP1 in Aβ Clearance” Section for a more detailed explanation.

LRP1 physiologically exists in two forms: a cell surface bound form (LRP1) and a truncated soluble form (sLRP1; Figure 1A). The surface-bound LRP1 is composed of two subunits that includes an extracellular α-chain (515 kDa) and a β-chain (85 kDa) containing a short extracellular extension, a single transmembrane domain and a 100 amino acid intracellular domain (Emonard et al., 2014). The characteristic motifs on the cytoplasmic tail of LRP1, namely, two NPXY motifs, one YXXL motif and two di-leucine motifs bind to endocytotic and scaffold adaptors, rapidly trafficking ligands like Aβ into endosomes in a clathrin-dependent manner (Li et al., 2001a; Deane et al., 2004, 2008) transporting them into the vascular lumen. Cell surface levels of LRP1 are controlled by proteolytic cleaving of its ectodomain. Several proteases including beta-secretase 1 (BACE-1), A Disintegrin And Metalloprotease (ADAM)-10, ADAM-12, ADAM-17, membrane type-1 matrix metalloprotease (MT1-MMP) and tissue plasminogen activator (t-PA) have been shown to be involved in shedding of LRP1 (Rozanov et al., 2004; von Arnim et al., 2005; Polavarapu et al., 2007; An et al., 2008; Liu et al., 2009; Selvais et al., 2011). The extracellular domain or ectodomain of LRP1 released after shedding is sLRP1 (Quinn et al., 1997), as described above. The truncated soluble form of LRP1 freely circulates in the plasma and sequesters unbound Aβ in the periphery (Sagare et al., 2007, 2013a). sLRP1 is also detectable in the cerebrospinal fluid (CSF; Qiu et al., 2001; Liu et al., 2009).

The endocytotic efficiency of LRP1 is dynamically regulated by post-translational phosphorylation of serine, tyrosine and threonine residues on its cytoplasmic tail (Bu et al., 1998; van der Geer, 2002), which also enables its downstream cell signaling functions. Phosphorylation on serine and tyrosine residues by protein kinase Cα (PKCα) enables the LRP1 intracellular domain to bind to adaptor protein Disabled-1 (Dab1) decreasing endocytosis by 25% (Ranganathan et al., 2004), whereas, phosphorylation on serine residues by protein kinase A (PKA) increases LRP1 endocytosis (Li et al., 2001b). Distinct from this, phosphorylation of tyrosine by *Src* activates the Ras signaling pathway rather than endocytosis (Barnes et al., 2003).

Interestingly, our recent study (Zhao et al., 2015) indicates that phosphatidylinositol binding clathrin assembly protein (PICALM; Dreyling et al., 1996; Tebar et al., 1999), a highly-validated risk factor for AD confirmed in several genome-wide association studies (Harold et al., 2009; Lambert et al., 2009; Carrasquillo et al., 2010, 2015; Chen et al., 2012; Tanzi, 2012; Liu et al., 2013; Morgen et al., 2014) binds to the intracellular tail of LRP1 at the YXXL domain and regulates endocytsosis of LRP1-Aβ complexes. Aβ binding to LRP1 presumably elicits a conformational change in its cytoplasmic tail, enabling PICALM-regulated endosomal transcytosis of Aβ (Zhao et al., 2015).

### Role of LRP1 in Aβ Clearance

In order to prevent pathological accumulations of Aβ in the brain, Aβ clearance from the cerebral milieu into periphery and out of the system is of prime importance. The multi-site role of the key Aβ-binding receptor, LRP1, helps eliminate systemic Aβ in a three-step serial clearance mechanism (Figure 1B; Zlokovic et al., 2010; Sagare et al., 2012). *Step 1* *(BBB)*. At the site of the BBB, surface LRP1 is expressed on the endothelial membrane facing the brain (abluminal). This membrane-bound LRP1 binds cerebral Aβ and rapidly initiates its clearance into the blood (luminal) side (Shibata et al., 2000; Deane et al., 2004, 2008; Ito et al., 2006; Bell et al., 2007; Sagare et al., 2007). Radiolabelled and unlabeled Aβ administered intracerebrally has appeared intact in plasma, indicating Aβ efflux from the brain by cerebrovascular LRP1 (Shiiki et al., 2004; Bell et al., 2007). *Step 2* *(Plasma)*. sLRP1 in the periphery contributes to Aβ clearance by sequestering free Aβ in circulation (Sagare et al., 2007, 2013a). Coimmunoprecipitation of sLRP1-bound Aβ in neurologically normal humans has indicated that circulating sLRP1 can sequester 70–90% of plasma Aβ (Sagare et al., 2007), thereby driving the Aβ gradient in favor of efflux across the BBB. This endogenous peripheral Aβ “sink” created by sLRP1 promotes Aβ clearance from the brain into circulation. *Step 3* *(Liver)*. LRP1 was first identified in the liver (Herz et al., 1988), and at the hepatic site, is responsible for binding to and clearing Aβ from the system (Tamaki et al., 2006; Sagare et al., 2011b; Sutcliffe et al., 2011; Sehgal et al., 2012). In addition to the liver, sLRP1-Aβ complexes are also removed by the kidney and spleen (Sagare et al., 2007).

## Recent Techniques to Study Aβ Clearance from the Brain

**Figure 2:**
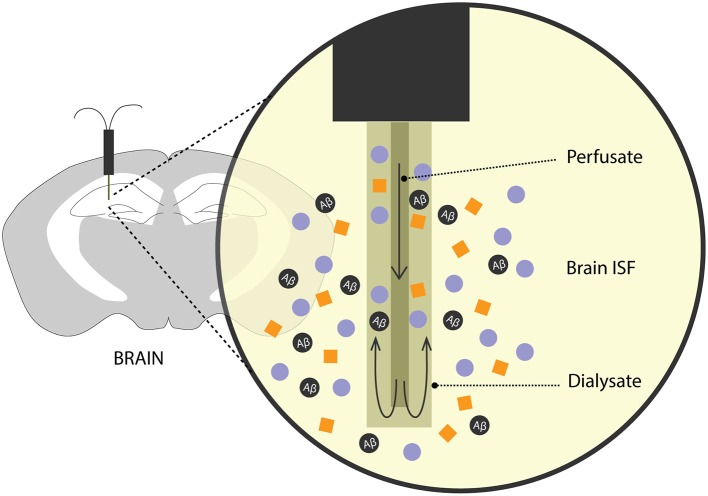
**Brain microdialysis.** A microdialysis probe is surgically implanted into a specific brain region of interest. Aβ and other solutes from the brain ISF freely enter the semipermeable membrane of the microdialysis probe. Fractions of microdialysates obtained over a period of time are then used to quantify Aβ levels reflecting steady-state levels and/or clearance of Aβ from the brain after inhibition of Aβ production.

For many years, methods involving intracerebral injection of radiolabelled Aβ and studying its efflux from the brain into the plasma and/or CSF has been a validated method to predict Aβ clearance. Over the recent years, there has been an increased interest in exploring the brain ISF pool for Aβ levels, its metabolic dynamics and clearance rates. To this effect, an analytical technique—microdialysis (Figure 2)—which has been popularly used to study neurotransmitters, has recently been recruited to study soluble Aβ in the brain of awake *APP* overexpressing mice (Cirrito et al., 2003; Chefer et al., 2009; Sagare et al., 2013b; Zhao et al., 2015).

In freely moving rodents, cerebral microdialysis enables continuous, time-lapsed readouts of Aβ levels in the brain ISF. Briefly, the microdialysis method entails implanting a small microdialysis probe (~ 200 μm in diameter) into a discrete brain region. Each probe has a characteristic recovery rate for the analyte of interest, here, Aβ, that is optimized *in vitro*. The probe comprises of a semipermeable membrane of a limited porosity, (in this case, 35 kDa) and is perfused with artificial CSF. Cerebral molecules up to 35 kDa in size can freely enter the membrane and are recovered through the microdialysate. Fractions of microdialysates can be collected over different periods of times to represent a neurochemical “snapshot” of the cerebral milieu around the probe along a certain time period. Aβ in the microdialysate is then quantified using biochemical assays like enzyme-linked immunosorbent assay (ELISA). In addition to obtaining steady-state measurements of Aβ, its efflux kinetics like half-life can be studied by administering a potent γ-secretase inhibitor, compound E which inhibits the production of soluble Aβ. The ISF Aβ levels from the microdialysate, upon compound E injection, now represent its clearance from the brain. This method has been used extensively in AD mouse models to obtain valuable information on genetic factors that affect clearance Aβ (Cirrito et al., 2003; Farris et al., 2007; Sagare et al., 2013b; Zhao et al., 2015). Intracerebral microdialysis has also been used to obtain human ISF Aβ concentrations in patients undergoing invasive intracranial monitoring (Brody et al., 2008).

A pioneering method was recently developed to measure rates of Aβ synthesis and clearance in human subjects. After intravenously infusing a stable isotope-labeled amino acid (^13^C_6_-leucine; Bateman et al., 2006; Mawuenyega et al., 2010) CSF and plasma were sampled over a 36-h period. The fractional synthesis rate (FSR) and fractional clearance rate (FCR) of *in vivo* Aβ was then quantified using high-resolution tandem mass spectrometry. This method was instrumental in demonstrating that diminished Aβ clearance, and not overproduction, contributes towards cerebral Aβ accumulation in late-onset AD (Bateman et al., 2006; Mawuenyega et al., 2010).

## Factors Regulating LRP1-Mediated Aβ Clearance

**Figure 3:**
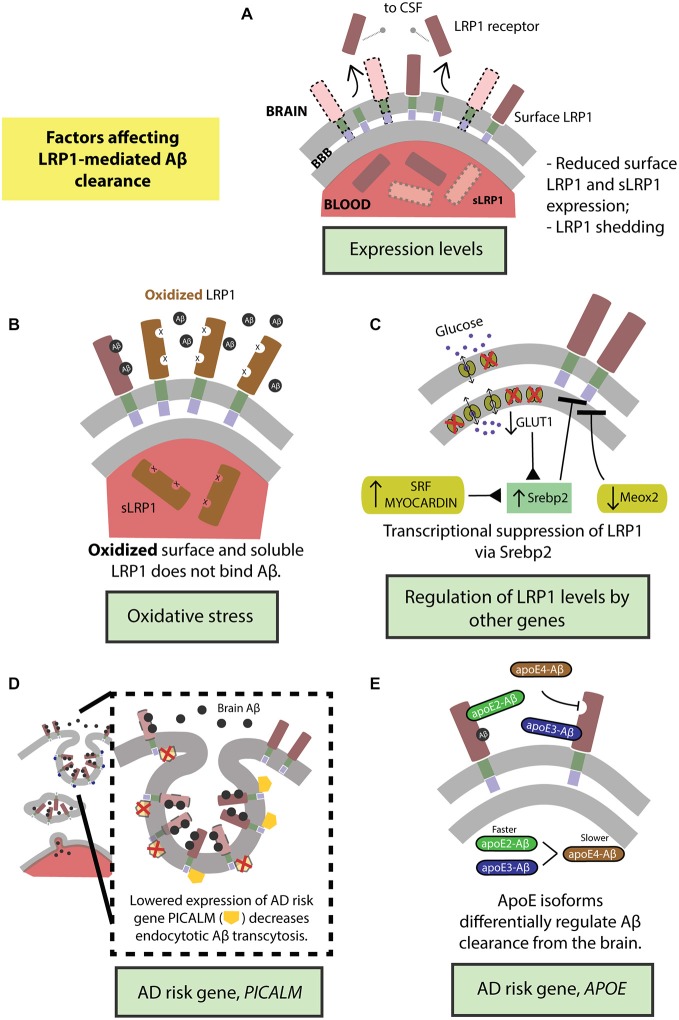
**Factors regulating transvascular LRP1-mediated Aβ clearance. (A)** Age-dependent reduction in LRP1 levels and its shedding and **(B)** oxidation of LRP1. LRP1 expression and Aβ clearance are further regulated by **(C)** transcriptional suppression by Srebp2 and *SLC2A1* (encoding glucose transporter, Glut1) in brain endothelial cells and SRF and MYOCD in vascular smooth muscle cells (VSMCs), as well as by *MEOX2* in brain endothelial cells. Moreover, well-known highly replicated Alzheimer’s disease (AD) genetic risk factors including *PICALM* **(D)** and *APOE4* gene **(E)** influence endocytosis and transcytosis of Aβ-LRP1 complexes across brain endothelium of the blood-brain barrier. See “Factors Regulating LRP1-Mediated Aβ Clearance” Section for a more detailed explanation.

The function of the BBB-localized LRP1 in actively removing Aβ from the brain is regulated by several factors. Fluctuations in LRP1 expression levels and structural modifications directly affect Aβ clearance. LRP1 expression is further regulated by several vascular genes in brain endothelium and VSMCs, and genetic risk factors for late-onset AD that can all modulate LRP1-mediated Aβ clearance (Figure 3).

### Expression Levels

LRP1 expression levels are significantly reduced in brain endothelial cells in normally aging and AD humans and animal models (Kang et al., 2000; Shibata et al., 2000; Deane et al., 2004; Donahue et al., 2006) leading to higher levels of Aβ in the brain (Figure 3A). Additionally, LRP1 levels are also diminished on VSMCs in AD patients (Bell et al., 2009). Importantly, there is a significant negative correlation between the expression of LRP1 on microvessels and Aβ accumulation in cerebrovasculature and brain parenchyma (Shibata et al., 2000; Donahue et al., 2006). Also, sLRP1 fraction bound to Aβ40 and Aβ42 is significantly reduced in Mild Cognitive Impairment (MCI) and AD subjects that corresponds to elevated levels of free Aβ40 and Aβ42 in plasma (Figure 3A; Sagare et al., 2011a). This free plasma Aβ can cross the BBB and re-enter the brain in a concentration-dependent manner mediated by the receptor for advanced glycation end products (RAGE), the major Aβ influx receptor at the BBB transporting Aβ from blood into the brain (Deane et al., 2003; Ujiie et al., 2003; Zlokovic, 2008). Interestingly, RAGE expression levels are increased in AD endothelium that is associated with cerebrovascular and brain accumulation of Aβ (Yan et al., 1996; Deane et al., 2003, 2012; Silverberg et al., 2010). Therefore, the accumulation of Aβ in the brain can be attributed to the cumulative effects of reduced expression of surface-bound LRP1 and sLRP1, causing higher cerebral and plasma Aβ levels respectively, and increased expression of RAGE, increasing the re-entry of Aβ into the brain.

#### Receptor Shedding

Lipoprotein receptors on the BBB are susceptible to ectodomain shedding which alters endocytotic transport and clearance of molecules, including Aβ, from the brain. Treating human brain endothelial cells with Aβ causes shedding of sLRP1 (Bachmeier et al., 2014; Figure 3A). Similarly, intracranial infusion of Aβ into mouse brain results in sLRP1 shedding (Bachmeier et al., 2014). In both the *in vitro* and *in vivo* experiments, the amount of sLRP1 shedding was reduced in the presence of ApoE2 or ApoE3 but not ApoE4 (Bachmeier et al., 2014). Furthermore, sLRP1 levels are increased in CSF from aged and AD subjects due to shedding (Qiu et al., 2001) caused by ADAM10 and ADAM17 (Liu et al., 2009). The dysregulation of shedded sLRP1, in part due to the presence of Aβ, could impair Aβ clearance from the brain and contribute to the pathogenesis of AD.

#### Oxidation

There is an increase in oxidative stress in the brain of aged and AD subjects (Moosmann and Behl, 2002; Chen and Zhong, 2014). This is evidenced by increased biomarker levels in the blood that reflect oxidative stress in the brain (Beal, 2005; Torres et al., 2011). Oligomeric Aβ induces oxidative stress that is believed to contribute to AD pathologies (Drake et al., 2003; Boyd-Kimball et al., 2006; Clementi et al., 2006). Importantly, LRP1 is oxidized in AD hippocampus and does not bind Aβ leading to increased Aβ deposition (Figure 3B; Drake et al., 2003). In addition to surface LRP1, in AD patients and animal models, sLRP1 binding to Aβ is disrupted by oxidation (Figure 3B; Sagare et al., 2012). Oxidized sLRP1, which does not bind to Aβ, is associated with elevated levels of free Aβ40 and Aβ42 in the plasma (Sagare et al., 2011a). Our previous studies have shown a significant positive correlation between CSF tau/Aβ42 ratios and oxidized sLRP plasma levels (Sagare et al., 2011a). Therefore, oxidation of both LRP1 and sLRP1 negatively impacts Aβ clearance from the brain. Moreover, there is likely a vicious cycle since Aβ induces LRP1 oxidation rendering the receptor less capable of clearing/reducing Aβ levels in the brain.

#### Regulation of LRP1 Expression by Genes in Vascular Cells

The expression of LRP1 is negatively regulated by its only known transcriptional suppressor; the sterol regulatory element binding protein (SREBP2; Llorente-Cortés et al., 2006, 2007). Recent studies have evidenced how Aβ clearance mechanisms in the CNS are indirectly altered by vascular- and metabolism-related genes via SREBP2-mediated regulation of LRP1 (Figure 3C).

##### LRP1 and GLUT1

The brain, which exclusively depends on the vasculature for essential metabolites, receives its supply of glucose across the BBB through the glucose transporter (GLUT1; encoded by *SLC2A1*). In AD, there is a reduction in this GLUT1 on cerebral microvessels (Mooradian et al., 1997). Diminished uptake of glucose, as studied by positron emission tomography (PET) using a glucose analog, 18F-2-fluoro-2-deoxy-D-glucose (FDG), is reported to be a forerunner to brain atrophy (Hunt et al., 2007), and has been observed in individuals with a genetic risk for AD, with a positive familial AD history, as well as those with none or mild cognitive deficits to eventually go on to develop AD (Hunt et al., 2007; Herholz, 2010).

Our group has recently demonstrated that GLUT1 deficiency in a transgenic AD mouse model overexpressing human *APP* Swedish mutant, *APP*^sw/0^, accelerated amyloid load and aggravated Aβ accumulation (Winkler et al., 2015). Incidentally, GLUT1 heterozygous mice (*Slc2a1*^+/−^) also expressed lower levels of LRP1 with respect to controls (*Slc2a1*^+/+^); the trend in diminished LRP1 expression further exacerbating with the addition of *APP* phenotype in *Slc2a1*^+/−^*APP*^Sw/0^ (Winkler et al., 2015). Re-expression and silencing of *Slc2a1* correspondingly boosted and decreased LRP1 levels, explaining the reversible nature of the GLUT1-LRP1 relationship; while suppressing LRP1 levels did not affect GLUT1 expression, indicating that GLUT1 acts upstream to LRP1. In deciphering the molecular mechanism behind this relationship, it was observed that *Slc2a1* deficiency upregulates the *SREBP2* transcription factor, in turn downregulating LRP1 expression (Figure 3C; Winkler et al., 2015).

Reductions in glucose transporters observed in AD (Kalaria and Harik, 1989; Horwood and Davies, 1994; Simpson et al., 1994; Mooradian et al., 1997), extends its effects beyond apparent hypometabolism to essentially affect Aβ clearance mechanisms by regulating LRP1 levels in the cerebral endothelia.

##### LRP1 and vascular-related genes

The expression levels of several vascular-related genes are altered in AD. For example, transcriptome profiling of human brain endothelial cells has indicated that the expression of mesenchyme homeobox gene 2 (*MEOX2*), a regulator of vascular differentiation and remodeling, is reduced in AD (Wu et al., 2005). The downregulation of *MEOX2*, and consequently the encoded protein, growth arrest-specific homeobox (GAX), is associated with altered angiogenesis, cerebral hypoperfusion and accumulation of brain Aβ (Wu et al., 2005). Unsurprisingly, *MEOX2* expression affects Aβ homeostasis by regulating LRP1 expression. Low levels of *MEOX2*, as studied *in vivo* and *in vitro* models, leads to diminished LRP1 levels at the BBB by promoting its proteosomal degradation (Figure 3C; Wu et al., 2005). On the other hand, two other interrelated transcriptional factors constituting the critical regulators of VSMCs differentiation, namely, serum response factor (SRF) and myocardin (MYOCD) are, in turn, upregulated in AD (Chow et al., 2007; Bell et al., 2009). The overexpression profile of SRF/MYOCD initiates a hypercontractile phenotype in the cerebral arteries through increased expression of SRF/MYOCD-regulated contractile proteins, thereby resulting in cerebral hypoperfusion, diminished neurovascular coupling and cerebral amyloid angiopathy (CAA; Chow et al., 2007). Cerebral VSMCs from AD patients with CAA exhibit, along with overexpressed SRF/MYOCD, an accumulation of Aβ and significantly lower levels of LRP in comparison with age-matched healthy controls (Bell et al., 2009). Following this, it was then observed that SRF/MYOCD overexpression in VSMCs transcriptionally regulates LRP1 levels by transactivation of SREBP2, diminishing LRP1 surface expression and affecting Aβ efflux from the brain (Figure 3C; Bell et al., 2009).

#### Regulation of LRP1-Mediated Aβ Endocytosis and Clearance by AD Risk Genes

##### PICALM

While the extracellular domain of LRP1 binds a diverse array of ligands, the intracellular cytoplasmic domain is actively involved in ligand endocytosis (Krieger and Herz, 1994; Reekmans et al., 2010). Recently, a gene crucial for endocytotic internalization of receptors—*PICALM* (Sorkin and von Zastrow, 2009; Treusch et al., 2011) encoding phosphatidylinositol binding clathrin assembly protein (Dreyling et al., 1996; Tebar et al., 1999), is a highly-validated risk factor for AD and has been confirmed in several genome-wide association studies (Harold et al., 2009; Lambert et al., 2009; Carrasquillo et al., 2010, 2015; Chen et al., 2012; Tanzi, 2012; Liu et al., 2013; Morgen et al., 2014). Interestingly, in our most recent work (Zhao et al., 2015), we observed that *Picalm* haploinsufficiency imparts diminished clearance of cerebral Aβ and accelerated amyloid pathology. Because the capillaries lining the BBB prolifically express PICALM (Baig et al., 2010; Parikh et al., 2014), we investigated whether this high-risk AD gene regulated the internalization of the main Aβ-clearance receptor, LRP1, thereby affecting Aβ clearance. Using primary human brain endothelial cells, we observed that fluorescently-labeled Aβ40-LRP1 complex rapidly colocalizes with PICALM, and remains associated with it for several minutes after exposure, indicating a downstream mechanism of PICALM-regulated clathrin-dependent endocytosis of Aβ40-LRP1 (Figure 3D). Further delineating the endocytotic fate Aβ40-LRP1, proximity ligation studies with several downstream players from endosomal pathway revealed that PICALM-driven Aβ40-LRP1 internalization is shunted away from lysosomal degradation and instead directed towards a transcytotic clearance pathway, shuttling Aβ from the brain into circulation (Figure 3D; Zhao et al., 2015). This seminal finding marks the first of its kind, mechanistically answering the integral question on the relation between the AD risk factor *PICALM*, amyloid load and LRP1 function.

The binding of PICALM to the intracellular tail of LRP1 at the YXXL domain is specific for Aβ as the ligand, and did not occur with other LRP1 ligands, like, apoE and α2-macroglobulin (A2M; Zhao et al., 2015). Aβ binding to LRP1 presumably elicits a conformational change in its cytoplasmic tail, enabling PICALM-regulated endosomal Aβ transcytosis. PICALM is downregulated in AD (Zhao et al., 2015); these reductions potentially contribute to an exacerbation in disease pathology by hindering LRP1-mediated Aβ transport, further tipping the Aβ balance in the brain (Figure 3D; Zlokovic et al., 2010; Sagare et al., 2012).

Importantly, single-nucleotide polymorphisms (SNPs) in *PICALM*, located upstream of the gene coding region but not in the coding region, have been identified to influence AD risk (Harold et al., 2009; Lambert et al., 2009; Carrasquillo et al., 2010, 2015; Chen et al., 2012; Tanzi, 2012; Liu et al., 2013; Morgen et al., 2014). It has been reported that some AD-associated SNPs influence *PICALM* expression (Raj et al., 2012). We recently studied the highly validated *rs3851179* *PICALM* variants whose *rs3851179^A^* allele is associated with a lower AD risk than the *rs3851179^G^* allele (Lambert et al., 2009, 2013) using inducible pluripotent stem cell (iPSC)-derived endothelial cells. These studies revealed that the protective *rs3851179^A^* allele significantly increased *PICALM* expression and, more importantly, Aβ clearance, reiterating the essential role of *PICALM* in Aβ clearance (Zhao et al., 2015).

##### ApoE

ApoE is a 34 kDa glycoprotein produced mainly by the glial cells in brain and liver in the periphery (Huang and Mahley, 2014). ApoE plays an important role in lipid metabolism (Mahley, 1988). In humans, the apoE exists in three isoforms, apoE2, apoE3 and apoE4 which differ from each other by either one or two amino acids at position 112 and 158 (Huang and Mahley, 2014). Several genome wide association studies in the past two decades identified *APOE4* as a major genetic risk factor for AD (Corder et al., 1993; Saunders et al., 1993; Tanzi, 2012). Recent studies suggest that apoE4 contributes to vascular and neuronal dysfunction via both Aβ-dependent and Aβ-independent pathways (Deane et al., 2008; Bell et al., 2012; Hudry et al., 2013; Huang and Mahley, 2014; Casey et al., 2015; Halliday et al., 2015). ApoE interacts with Aβ and plays an important role in its metabolism and AD pathogenesis (Wisniewski and Frangione, 1992; Holtzman et al., 2000; DeMattos et al., 2004; Bell et al., 2007; Deane et al., 2008; Jiang et al., 2008; Castellano et al., 2011; Zlokovic, 2013; Tai et al., 2014). Previous work from our group has shown that lipidation status of apoE can affect its binding to Aβ and clearance across the BBB (Martel et al., 1997; Bell et al., 2007; Deane et al., 2008). Our studies have also shown that rapid BBB clearance of Aβ complexed to apoE2 and apoE3 occurs mainly by LRP1 (Figure 3E), by contrast, apoE4-Aβ complexes are removed by slower very low density lipoprotein receptor (VLDLR)-mediated internalization and transcytosis (Bell et al., 2007; Deane et al., 2008).

## Restoration of LRP1

In addressing the reduced expression of LRP1 on AD capillaries, a potential method of selectively targeting LRP1 for its restoration in aging or in diseased state is via the delivery of gene transfer vectors. Viral-mediated gene transfer methods, especially adeno-associated viral (AAV) systems have proven to be effective both in different peripheral cell types as well as in the CNS (Davidson et al., 2000; Mingozzi and High, 2011), are safe and more commonly used for targeted gene therapy. In general, gene therapy directed towards neurological disorders remains challenging due to the restricted entry of vectors into the brain authorized by the BBB endothelial lining. However, targeting a receptor like LRP1 is more achievable due to its favorable position on the endothelial membrane, making it directly accessible to therapeutic interventions that may be administered intravenously. In animal models, vascular endothelial cells have been successfully transduced with AAV-2 vector system by using peptides with a high affinity for cerebral vasculature, specific for normal and diseased states identified by *in vivo* phage panning (Chen et al., 2009). Recently, AAV-9, a serotype less affected by human neutralizing antibodies, has been developed for a highly efficient transduction of endothelial cells (Varadi et al., 2012). Using these recently-developed gene transfer techniques offering a vascular-directed biodistribution, it is conceivable to use AAV-based targeting vectors to deliver LRP1 whole cDNAs or a part of its domains to restore reduced LRP1 expression at the BBB in AD.

Additionally, another candidate site for restoration or selective enhancement of LRP1 by gene therapy is the liver (Sagare et al., 2012). Restoring normal LRP1 levels in hepatocytes by use of the highly successful liver-directed AAV-based gene transfer methods (Mingozzi and High, 2011; Wang et al., 2011) can systemically “vacuum” out the peripheral Aβ and, in turn, promote the removal of brain Aβ by driving the Aβ gradient.

Moreover, Aβ clearance therapy is feasible at the peripheral “sink” constituent of Aβ homeostasis (Zlokovic et al., 2010; Sagare et al., 2011b, 2012, 2013a). In fact, circulating sLRP1-Aβ in plasma serves as an early biomarker for mild cognitive impairment preceding AD-type dementia (Sagare et al., 2011a). Soluble LRP1 and/or its wild-type recombinant cluster IV, WT-LRPIV, bind to free Aβ, preventing it from reentering the brain via the RAGE receptor, and therapeutically enabling reductions in Aβ pathology (Sagare et al., 2007). In order to limit the binding of the recombinant LRPIV to only the neurotoxic Aβ while excluding other LRP-binding ligands, a mutant of LRPIV was recently developed, exhibiting higher binding affinity to Aβ (Sagare et al., 2013a). This mutant, LRPIV-D3674 effectively replaced oxidized sLRP1, is 25–27% more effective than WT-LRPIV in clearing brain endogenous Aβ, and within 3 months of treatment, significantly reduced Aβ levels in hippocampus and cortex of *APP*^sw/0^ mice (Sagare et al., 2013a). LRPIV-D3674 is thus capable of regulating Aβ levels in the periphery, and forms the rationale for an efficient Aβ clearance therapy. Restoring reduced or oxidized sLRP1, that contributes to ~70% of bound Aβ (Sagare et al., 2007), can restore the natural peripheral “sink” and salvage the irregular Aβ homeostasis in AD.

In developing these LRP1-targeted efforts, it is, however, important to exert caution in Aβ specificity and the safety of the therapies as LRP1 has a multi-functional role as an endocytotic and a cell signaling receptor in several other systemic mechanisms.

## Conclusion

AD is a growing epidemic. There are currently 5.2 million Americans suffering from this disease and the number of individuals affected is projected to triple by the year 2050 (Sano et al., 2013). Failure in Aβ clearance is highly recognized in relation to AD pathogenesis in sporadic or late-onset AD, the most common form of AD occurring in over 95% of patients (Tanzi, 2012). Here, we have briefly reviewed the transvascular Aβ clearance from the brain mediated by LRP1.

Because the cerebrovasculature highly influences Aβ homeostasis it would be beneficial to develop therapeutic strategies in AD including both, the neuronal and vascular components of the CNS. In addition to the advantage that the vascular system is easily accessible from the periphery for pharmacological and genetic manipulations, vascular damage patterns typically precede neuronal injury, providing an opportunistic window for treatment—a concept proposed in the vascular two-hit hypothesis (Zlokovic, 2011). According to the vascular two-hit hypothesis, an initial vascular insult to the brain (*hit 1*) elicited by hypoxia, hypoperfusion or a disrupted BBB precedes observed amyloid pathology in AD. Accumulated Aβ (*hit 2*), predominantly an effect of faulty Aβ clearance in late-onset AD, now triggers a pathological cascade of neuronal injury, cognitive decline and AD-dementia.

LRP1 plays a regulatory role in both, the *hit 1* (before Aβ accumulation) as well as the *hit 2* (amyloid pathology) phases of the AD disease progression, making it an invaluable target in AD clearance therapy (Zlokovic et al., 2010). Currently, there are no drugs that can prevent or reverse AD. Targeted LRP1-based therapies in restoration/enhancement of surface LRP1 at the BBB or on hepatocytes, or the peripheral application of LRPIV Aβ-binding clusters are currently being explored, and show potential in serving as Aβ clearance therapies (Sagare et al., 2012, 2013a). Due to the numerous factors like oxidative stress, receptor shedding, and the influence of other genes, among others, that regulate LRP1 expression and function, thereby affecting Aβ clearance, there is a compelling need to adapt a multifaceted approach in addressing therapeutic interventions. Pharmacological interventions may be used in conjunction with gene therapy; for example, administration of withanolides and withanosides reverse AD pathology in *APP/PS1* mice by enhancing LRP1 in the brain microvessels and liver (Sehgal et al., 2012), olive-oil-derived oleocanthal enhances cerebral Aβ clearance by upregulating LRP1 at the BBB (Abuznait et al., 2013). Moreover, changes in lifestyle can have a meaningful impact as preventive measures in AD. An antioxidant-rich diet, such as the recently investigated Mediterranean-DASH (Dietary Approaches to Stop Hypertension) Intervention for Neurodegenerative Delay (MIND) diet has shown potential in reducing AD risk (Morris et al., 2015), presumably by rousing the body’s natural anti-oxidant defense mechanism. Incorporating physical activity into one’s lifestyle serves as a pro-angiogenic factor having beneficial effects on cognition. Additionally, another promising compound performing as a vasculoprotectant, antioxidant and an anti-inflammatory agent is the activated protein C (APC; Griffin et al., 2002, 2015).

AD-associated genes regulating LRP1 function, like *APOE, SLC2A1* and *PICALM*, too extend potential as targets for AD therapy in alleviating AD pathology and/or amend Aβ imbalance. Future studies focused on genetically engineered mice possessing variants in these AD risk genes can generate more knowledge on the role of LRP1-mediate transvacular clearance of Aβ.

## Conflict of Interest Statement

The authors declare that the research was conducted in the absence of any commercial or financial relationships that could be construed as a potential conflict of interest.
